# Structure and Anti-Inflammation Potential of Lipoteichoic Acids Isolated from *Lactobacillus* Strains

**DOI:** 10.3390/foods11111610

**Published:** 2022-05-30

**Authors:** Qianqian Lu, Yingqi Guo, Guo Yang, Lei Cui, Zhen Wu, Xiaoqun Zeng, Daodong Pan, Zhendong Cai

**Affiliations:** 1 Key Laboratory of Animal Protein Deep Processing Technology of Zhejiang Province, College of Food and Pharmaceutical Sciences, Ningbo University, Ningbo 315800, China; fm08152022@163.com (Q.L.); guoyingqi1998@163.com (Y.G.); yg1227936963@163.com (G.Y.); cuilei0114@163.com (L.C.); wuzhen@nbu.edu.cn (Z.W.); zengxiaoqun@nbu.edu.cn (X.Z.); daodongpan@163.com (D.P.); 2 State Key Laboratory for Managing Biotic and Chemical Threats to the Quality and Safety of Agro-Products, Ningbo University, Ningbo 315211, China

**Keywords:** *Lactobacillus*, lipoteichoic acid, adhesion, anti-inflammation, MAPK and NF-κB signals

## Abstract

*Lactobacillus* are normal inhabitants of the gastrointestinal tract and confer a variety of health effects. Lipoteichoic acid (LTA), an amphiphilic substance located in the cell membrane, is a key molecule in probiotic–host crosstalk. Through the characterization of structural characteristics of LTA molecules derived from *Lactobacillus plantarum* A3, *Lactobacillus reuteri* DMSZ 8533, and *Lactobacillus acidophilus* CICC 6074, there exists some heterogeneity in LTA molecules, which perhaps contributes to the distinguishable adhesion properties of *Lactobacillus* strains based on fluorescence microscopy observations. In LPS-induced RAW 264.7 cells, LTAs derived from three *Lactobacillus* strains obviously alleviated inflammatory responses as evidenced by the altered inflammatory cytokine levels of TNF-α, IL-6, and IL-10. Western blotting demonstrated that *L. reuteri* LTA blocked LPS-triggered expression of the MAPK and NF-κB pathways. The findings further validated that LTA is an important effector molecule and deserves further consideration as an alternative therapeutic for ulcerative colitis treatment.

## 1. Introduction

The immunomodulatory function of probiotics has gained more attention due to prophylactic and therapeutic functions in a variety of disorders [1]. Lactic acid bacteria are normal inhabitants of the gastrointestinal tract of the host and play important roles in maintaining intestinal homeostasis [2]. Probiotic–host interactions are mediated by surface located molecules, known as microbe-associated molecular patterns (MAMPs), such as surface layer proteins SlpAs, peptidoglycan, sortase-dependent proteins, and myosin-cross reactive proteins, which can be recognized by pattern recognition receptors (PRRs) and produce strain-specific health effects on the host [3,4].

Teichoic acid (TA) is a second major component of cell walls in gram-positive bacteria [5]. As a major component of TA, lipoteichoic acid (LTA), an amphiphilic polycondensate, anchors glycolipids into the cell membrane and transects the peptidoglycan layer extending into the cell envelope [6]. The glycolipid mostly comprises glycerol phosphate (GroP) repeating units, Glc(β1-6)-Glc(β1-3) diacylglycerol, with poly-GroP chains covalently linked to glycolipids by hexose residues [6]. The free hydroxyl groups on the poly-GroP can be modified by glucose, galactose, D-Alanine (D-Ala), or N-acetylglucosamine (GlcNAc) [7]. Notably, LTAs share many pathophysiological properties that are comparable to those observed for lipopolysaccharides (LPSs) in pathogenic bacteria [8]. As reported, LTA isolated from *Streptococcus pyogenes*, *Streptococcus pneumonia*, and *Staphylococcus aureus* has been demonstrated to induce the expression of inflammatory cytokines and mediators [9,10]; that is, they are coincident with other LTAs derived from pathogens, which can activate macrophages and then release various cytokines [11,12]. Therefore, LTA is a major virulence factor of pathogenic bacteria. Unlike LTAs in the pathogenic bacteria, LTAs have a beneficial effect on the host’s immune system in probiotic bacteria [13]. For example, *Clostridium butyricum* LTA obviously alleviates inflammation and apoptosis caused by *S. aureus* LTA in human colon cancer HT-29 cells [14]. *Lactobacillus plantarum* LTA attenuates a TNFα-induced inflammatory response in HT-29 cells through the inhibition of MAPK (mitogen-activated protein kinase) and NF-κB (nuclear factor kappa B) signaling [15], indicating that LTAs from probiotics have a protective role in the host’s immune system.

However, LTAs possess differential immune regulatory capacities in lactobacilli. As reported, both LTAs isolated from *L. plantarum* and *Lactobacillus sakei* can inhibit endotoxin-mediated inflammation, while they cause differential effects in cytokine production and the Th1/Th2 immune response [16]. Likewise, LTAs show the distinguishable capacity to inhibit poly I:C-induced IL-8 levels in *Lactobacillus delbrueckii*, *L. sakei*, *Lactobacillus rhamnosus GG*, and *L. plantarum* LTA [17]. A recent study has proposed that LTAs provoke differential immunostimulatory responses in four *Lactiplantibacillus plantarum* strains [18], suggesting that LTAs perhaps have strain-specific properties and effects on the immune response. To further investigate immunomodulatory properties of *Lactobacillus* LTA, *L. plantarum*, *Lactobacillus reuteri*, and *Lactobacillus acidophilus* were selected to assess the function of LTA in LPS-induced RAW 264.7 cells. Through multi-spectrometric analyses, LTA molecules exhibited a similar basic structure harboring some strain-specific differences in lactobacilli, which perhaps resulted in differential adhesion of *Lactobacillus* strains in the Caco-2 cell model. Western blotting demonstrated that *L. reuteri* LTA is a key effector molecule that showed strong anti-inflammatory properties in LPS-stimulated RAW 264.7 cells.

## 2. Materials and Methods

### 2.1. Reagents

Man Rogosa Sharpe (MRS) was obtained from Shanghai Yuanye Biological Technology Co., Ltd. (Shanghai, China). Octyl-Sepharose column CL-4B, DEAE-52 cellulose, fetal bovine serum (FBS), fluorescein and isothiocyanate (FITC), and Hoechst 33342 were purchased from Shanghai SolarBio Bioscience and Technology Co., Ltd. (Shanghai, China). The standard LTA from *S. aureus* was obtained from Sigma-Aldrich (L2515). RAW 264.7 macrophage and Caco-2 cells were obtained from the National Collection of Authenticated Cell Cultures (Shanghai, China). The cell counting kit-8 (CCK-8) and penicillin-streptomycin solution (P/S) were obtained from Beyotime Biotechnology (Shanghai, China). ELISA Kits of TNF-α, IL-10, and IL-6 were purchased from R&D Systems Europe Ltd. (Abingdon, UK). Antibodies for β-actin, p44, p-p44, JNK, p-JNK, p65, and p-p65 were obtained from Cell Signaling Technology. Other reagents were purchased from Nanjing Jiancheng Bioengineering Institute (Nanjing, China).

### 2.2. Adhesion Ability of Lactobacillus Strains

The adhesion assay on the Caco-2 cells was carried out as previously described with some modifications [19,20]. *L. plantarum* A3, *L. reuteri* DMSZ 8533, and *L. acidophilus* CICC 6074, which were kept in our laboratory, were grown in MRS broth, harvested, washed, and resuspended in sterile PBS and then adjusted to OD of 1 at 600 nm. Bacteria were dissolved in 0.05 mg mL^−1^ FITC for 1 h under dark conditions (37 °C) and then washed repeatedly with PBS (7.2) for FITC-labeled strains preparation. Caco-2 cells were grown in a DMEM medium supplemented with 20% FBS and 1% P/S under an atmosphere of 5% CO_2_ at 37 °C. As Caco-2 cells cultured in about 60 % of the wells, FITC-labeled strains were co-incubated with Caco-2 cells for 2 h. Cell samples were then washed repeatedly with PBS (pH 7.2) to remove the excess stain. A volume of 4% paraformaldehyde was used for fixing the samples for 20 min, and the nucleus was stained with Hoechst 33342 (0.1 mg mL^−1^) for 15 min. The fixed cells were photographed by an inverted fluorescence microscope.

### 2.3. Extraction of LTA

*Lactobacillus* LTAs were extracted and purified as previously described [21,22]. Briefly, 10 g of the bacteria pellets was dissolved in 50 mL of sodium citrate buffer (0.1 M, pH 4.7). Under the conditions of an ice-cold bath, the solution was ultrasonicated for 30 min. Then, the suspension was mixed with 50 mL of n-butyl alcohol and stirred for 2 h using a magnetic mixer. After phase separation by centrifugation, the LTA-containing aqueous phase was collected, dialyzed against pure water overnight and further lyophilized. The crude extraction was separated with octyl agarose gel CL-4B, which was pre-balanced with 15% n-propanol in 0.1 M ammonium acetate solution (pH 4.7) and then eluted with linear 15–60% n-propanol in 0.1 M ammonium acetate buffer. The eluent containing LTA was dialyzed overnight and then separated by the DEAE-52 cellulose column pre-equilibrated with 0.1 M ammonium acetate solution (pH 4.7). Fractions were dialyzed in pure water for 48 h and then lyophilized for further use.

### 2.4. Structural Analysis of LTA

The structure of LTA was identified by Fourier transform infrared spectroscopy (FT-IR) analyses and nuclear magnetic resonance (NMR) spectrometry according to previous methods [22,23]. After the standard LTA of *S. aureus* and those from *Lactobacillus* were fully mixed with KBr, ground, and pressed. The infrared spectrum analysis was carried out at a wavelength range of 750–3750 cm^−1^. The 15 mg samples were dissolved in 0.5 mL dd H_2_O and tested on NMR apparatus for ^1^H.

### 2.5. Cell Viability Assay

The roles of LTAs on the viability of RAW 264.7 cells were examined through CCK-8 kit assay. RAW 264.7 cells (1 × 10^4^) were seeded on 96-well plates and then incubated for 12 h. The cells were supplemented with 100 μL LTA (0, 25, 50, and 100 μg mL^−1^) for 18 h. After that, each well was supplemented with 10 μL CCK-8 reagents and incubated for another 6 h. The absorption of the reaction solution was detected at a wavelength of 450 nm using a Synergy microplate reader. The viability of the RAW 264.7 cells was calculated as follows:

$$\mathrm{Cell}\;\mathrm{viability}=\frac{\left(A-C\right)}{\left(B-C\right)}\times 100\%$$

where A represents the holes with cells with CCK-8 and LTA added, B represents the holes with cells with CCK-8 but not LTA added, and C represents the holes without cells where CCK-8 and LTA was added.

### 2.6. Cytokine Measurements

As RAW 264.7 cells reached 80% confluence in the 96-well plates, the cells were supplemented with LPS (500 ng mL^−1^) for 10 h, followed by the supplementation of *Lactobacillus* LTA with a final concentration of 0, 25, 50, and 100 μg mL^−1^ for another 18 h, respectively. Supernatants of each treatment were collected after centrifugation at 10,000 rpm at 4 °C for 10 min. The cytokines of IL-6, TNF-α, and IL-10 in the cell supernatants were detected using the ELISA kits. The absorption was detected at 450 nm using a microplate reader (Thermo Fisher Scientific, Waltham, MA, USA).

### 2.7. Western Blotting Analysis

The lysates of each treatment were prepared as previously described [24]. Protein concentration was detected through a bicinchoninic acid (BCA) method. Total proteins (equal amounts) were separated by 12% SDS-PAGE and then transferred to polyvinylidene difluoride (PVDF) membranes. The PVDF membranes were subsequently blocked with 5% bovine serum albumin (BSA) for 2 h. Next, the membranes were respectively incubated with primary antibodies of JNK, p-JNK, ERK, p-ERK, p65, p-p65, and β-actin in Tris-buffered saline/Tween 20 (TBS-T) overnight at 4 °C, which were then incubated with horseradish peroxidase (HRP)-conjugated secondary antibodies for 1 h. Immunoreactive bands were visualized using ChemiScope 6000 Exp image system (CliNX, Shanghai, China). Finally, Image J software (National Institutes of Health) was used to analyze the gray value of each strip.

### 2.8. Statistical Analysis

Data were presented as mean ± SD of three biological repetitions and then statistically analyzed using IBM SPSS software 25 (IBM Corp., Armonk, NY, USA). Statistical differences were determined through one-way ANOVA followed by Duncan’s post-hoc test (*p* < 0.05).

## 3. Results

### 3.1. Structural Properties of Lactobacillus LTA

**Figure 1:**
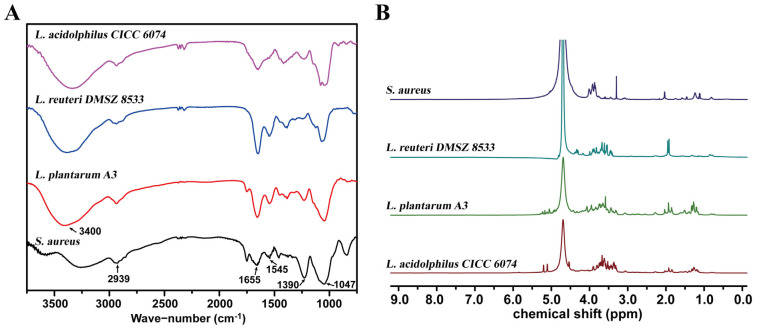
Multi−spectrometric analyses of the LTA structure in *Lactobacillus* strains. (**A**) The FT−IR spectra of LTA from *Lactobacillus* and LTA standard substance of *S. aureus*. (**B**) NMR spectra of intact LTAs. ^1^H NMR spectra (500 MHz, 298 K) of intact LTAs from *S. aureus* and *Lactobacillus* in 2H_2_O.

To characterize the structural characteristics of *Lactobacillus* LTA, LTA samples from *L. reuteri* DMSZ 8533, *L. acidophilus* CICC 6074, and *L. plantarum* A3 were detected by the FT-IR at a scanning wavelength range of 750–3750 cm^−1^. As shown in Figure 1A, an -OH group’s stretching vibration was visible at 3400 cm^−1^. The peak at 2939 cm^−1^ ascribed the vibration of -CH bonding inside of the fatty acid chains. The weak bands appearing near 1655 cm^−1^ and 1545 cm^−1^ were attributed to the C=O and N-H bonds of α-D-N-acetylglucosamine, respectively. Furthermore, the other adsorption band of the νs (COO^−^) of the alanine was visible at 1390 cm^−1^. The peak near 1047 cm^−1^ suggested the P-O stretching vibration. Notably, both LTAs from *L. plantarum* and *L. reuteri* presented an obvious N-H vibration peak at 1545 cm^−1^, while it was absent in *L. acidolphilus* LTA, indicating that LTA molecules of *L. reuteri* DMSZ 8533 and *L. plantarum* A3 no longer had saturated acyl fatty acid chain modifications.

High-field 500 MHz NMR spectrometry was applied to the LTA analysis. As shown in (Figure 1B), the ^1^H NMR of the LTA standard substance of *S. aureus* displayed four singlets at 2.04, 1.24, 1.11, and 0.81 ppm that were attributed to different CH-groups of alanine, which was indicative of the alanine modification structure. In the ^1^H NMR spectrum of *L. reuteri* LTA, there was a distinguishable set of ^1^H resonance represented by the characteristic singlet at 1.93 and the broad signal at δH 4.38–4.28 ppm. For *L. plantarum* LTA, the ^1^H NMR spectrum demonstrated that major differences were at δH = 1.85 and 4.91–5.25 (Figure 1B). Among them, the most significant difference was observed by the chemical shift at δH = 5.21 and 5.11 in *L. acidophilus* LTA. The ^1^H NMR spectra of LTA samples were complicated due to the plentiful peak overlaps in these samples. Combined with previous studies [6,22], it was postulated that the three *Lactobacillus* strains had significant differences in the length and saturation of α-D-N-acetylglucosamine, alanine, and acyl fatty acid chains.

### 3.2. Adhesion Properties of Lactobacillus Strains to Caco-2 Cells

**Figure 2:**
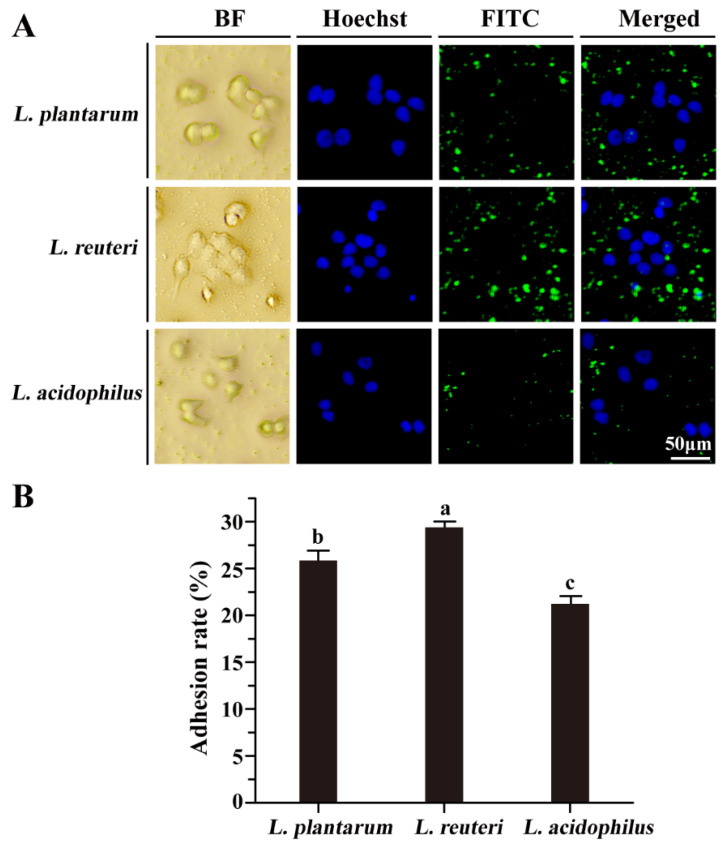
Adhesion of *Lactobacillus* on Caco-2 cells after 2 h of incubation at 37 °C in a 5% CO_2_ atmosphere. (**A**) *Lactobacillus* was stained with FITC (green fluorescence), and the nuclei of Caco-2 cells were stained with Hoechst 33342. (**B**) The adhesion rate was evaluated by the fluorescence intensity of the *Lactobacillus* strains. Values with the different letters are significantly different, while the same letters indicate no significant difference among the tested strains (Duncan’s test, *p* < 0.05).

To investigate the adhesion capacity of *L. reuteri* DMSZ 8533, *L. acidophilus* CICC 6074, and *L. plantarum* A3, all three strains were individually labeled with FITC and then co-incubated with Caco-2 cells for 2 h at 37 °C. FITC-labeled strains of *L. reuteri* and *L. plantarum* showed dramatically increased adhesion with strong intensities of fluorescence signals compared to that of *L. acidophilus* (Figure 2A). Quantitative analysis further validated that both *L. reuteri* (29.39 ± 0.62%) and *L. plantarum* (25.84 ± 0.9%), especially *L. reuteri*, exhibited significantly higher adhesion rates compared to *L. acidophilus* (21.24 ± 0.84%) based on the fluorescence microscopy observations (Figure 2B), suggesting that *Lactobacillus* might have distinguishable adhesion properties.

### 3.3. The Inhibitory Effects of LTAs on the Secretion of Inflammatory Cytokines in RAW 264.7 Cells

**Figure 3:**
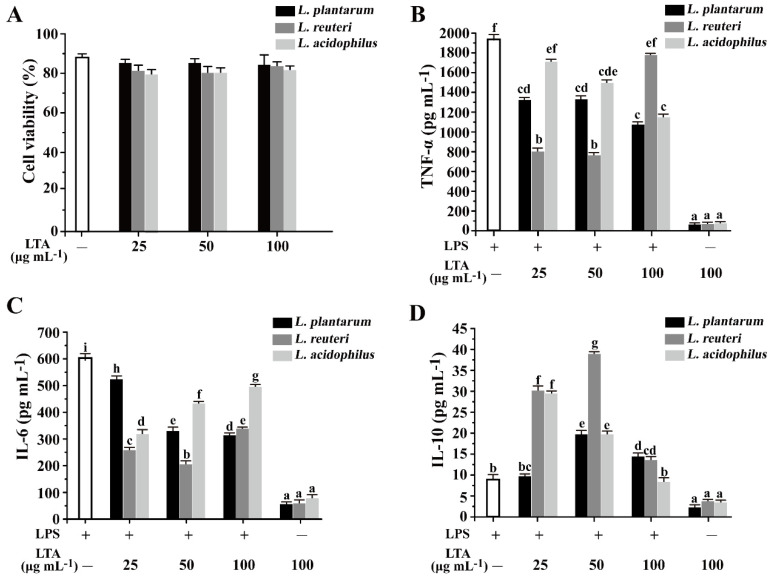
Effects of LTA on cytotoxicity and cytokines TNF-α, IL-10, and IL-6 expression in LPS-stimulated RAW264.7 cells. (**A**) RAW 264.7 cells were supplemented with different concentrations of LTA, and cell viability was determined by CCK-8 kit assay. (**B**) Levels of TNF-α were measured by ELISA. (**C**) Levels of IL-6 were measured by ELISA. (**D**) Levels of IL-10 were measured by ELISA. Values with different letters are significantly different, while same letters indicate no significant difference among the tested strains (Duncan’s test, *p* < 0.05). “+” and “−” mean RAW264.7 cells treated with or without additional LPS or LTA.

Prior to experimentation, the cytotoxicity of LTA levels was validated using a CCK-8 kit. As can be seen from Figure 3A, *Lactobacillus* LTAs of *L. reuteri* DMSZ 8533, *L. acidophilus* CICC 6074, and *L. plantarum* A3 had no obvious effects on cell viability and showed no significant differences at the tested concentrations of 25, 50, and 100 μg mL^−1^ compared with that of no LTA treatment, which suggested that no cytotoxic effects were observed for up to 100 μg mL^−1^ LTA from all *Lactobacillus* strains. Next, the effects of *Lactobacillus* LTA on LPS-induced RAW 264.7 cells were evaluated using ELISA kits. Compared to the treatment of LTA alone, LPS significantly provoked the secretion of TNF-α and IL-6 (Figure 3B,C). While LTAs isolated from the three strains significantly inhibited LPS-induced TNF-α production, especially for *L. plantarum* and *L. acidophilus*, LTAs that were dose-dependent decreased LPS-induced production of TNF-α (Figure 3B). For the *L. reuteri* LTA treatment, there existed a significant inhibitory expression of TNF-α at lower concentrations of 25 and 50 μg mL^−1^ (Figure 3B).

Similarly, LTAs from all three strains caused an obvious decrease in the production of IL-6 (Figure 3C). By contrast, IL-10, an anti-inflammatory cytokine, was activated by LTAs from the three strains in certain conditions (Figure 3D). Compared to that of the other two strains, *L. reuteri* LTA significantly increased the production of IL-10 at the tested concentrations, especially at 25 and 50 μg mL^−1^ (Figure 3D). Therefore, *Lactobacillus* LTAs played a crucial role in modulating both pro- and anti-inflammatory cytokines in LPS-induced RAW 264.7 cells.

### 3.4. Anti-Inflammation of LTA Involved in MAPK/NF-κB Signaling Pathways

**Figure 4:**
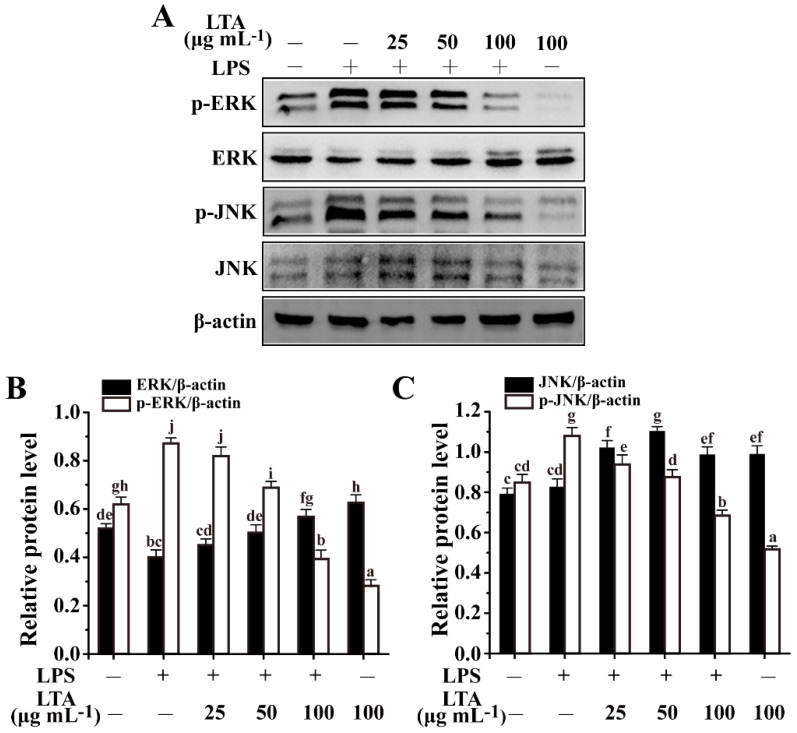
LTA attenuated MAPK signaling in LPS-induced RAW264.7 cells. (**A**) Phosphorylation of ERK and JNK MAPK was detected through Western blotting analysis. (**B**) Relative protein levels meant the phosphorylation of ERK expression was relative to β-actin and ERK expression was relative to β-actin. (**C**) Relative protein levels meant the phosphorylation of JNK expression was relative to β-actin and JNK expression was relative to β-actin. Values with different letters are significantly different, while same letters indicate no significant difference among the tested strains (Duncan’s test, *p* < 0.05). “+” and “−” mean RAW264.7 cells treated with or without additional LPS or LTA.

Combining data of adhesion and cytokines, *L. reuteri* LTA was selected as a representative to characterize the underlying mechanism of LTA-mediated immunomodulatory activity. MAPK families (ERK and JNK) and NF-κB (p65 and p50 subunits) have been extensively investigated in LPS-stimulated RAW 264.7 cells [3,25]. Here, LPS caused a significant increase in the phosphorylation of both ERK and JNK MAPK compared with the control group without any treatments, while both of them were dramatically attenuated by LTA in a dose-dependent manner (Figure 4A–C). In comparison, LTA alone had no obvious effects on the phosphorylation of both ERK and JNK MAPK.

**Figure 5:**
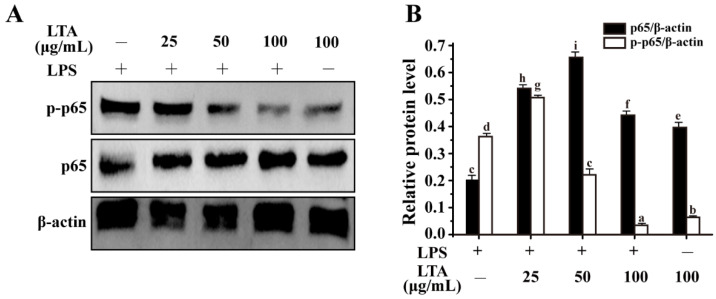
LTA suppresses NF-κB signaling in LPS-induced RAW264.7 cells. (**A**) Phosphorylation of p65 was detected by Western blotting. (**B**) Relative protein levels meant the phosphorylation of p65 expression was relative to β-actin and p65 expression was relative to β-actin. Values with the different letters are significantly different, while same letters indicate no significant difference among the tested strains (Duncan’s test, *p* < 0.05). “+” and “−” mean RAW264.7 cells treated with or without additional LPS or LTA.

NF-κB (p65 and p50 subunits) is a distinct downstream signaling molecule for MyD88 signaling, which eventually modulates the expression of cytokine genes [3]. As shown in Figure 5A,B, LPS significantly up-regulated phosphorylation of p65, while LPS-induced phosphorylation of p65 was obviously alleviated by *L. reuteri* LTA in a concentration-dependent manner. Collectively, a similar inhibitory trend occurred regarding the phosphorylation of both MAPK and NF-κB signaling. The findings further supported the hypothesis that MyD88-dependent MAPK and NF-κB pathways play crucial roles in LTA-mediated anti-inflammation.

## 4. Discussion

Lactic acid bacteria have gained substantial attention in maintaining intestinal homeostasis [26]. For a new probiotic strain, stable adhesion to the intestinal epithelium is one of the crucial criteria for evaluating probiotic action, which contributes to the competition between probiotics and pathogenic bacteria for the adhesion to intestinal epithelium [19]. Extensive studies have demonstrated that adhesion characteristics of lactobacilli are correlated with molecular effectors at the cell surface. As a membrane-associated amphiphile adhesin, LTA has been broadly demonstrated to be a mediator of the adhesion of *Lactobacillus* to human epithelial cells [5]. In addition, research has demonstrated the immunomodulatory properties of *Lactobacillus* LTAs [15,16], while relatively little is known about the difference of immunostimulatory capacity of LTAs isolated from *L. plantarum*, *L. reuteri*, and *L. acidophilus*; therefore, it is of great importance to further unmask the immunomodulation of LTAs of these three *Lactobacillus* strains. Our data found that *L. reuteri* had significantly higher adhesion properties compared to the other two strains. Most importantly, *L. reuteri* LTA exhibited a strong immunomodulatory capacity in the secretion of inflammatory cytokines among these three *Lactobacillus* strains. Our findings supported the hypothesis that LTAs have strain-specific properties and effects on the immune response in lactobacilli, which perhaps involves in the distinctive structural features of their LTAs. Based on comparative analysis, *L. reuteri* LTA deserves further consideration as an alternative therapeutic for ulcerative colitis (UC) treatment.

Lactobacilli expressing LTA stimulate the host’s immune system against pathogen competition for specific receptors [27]. As reported, LTAs can cause differential immunostimulatory activity due to the heterogeneity in their structures [6]. This was corroborated by other reports that the link between the structure and immunomodulation of LTA varied greatly among bacteria [28]. To further characterize the immunostimulatory activity of *Lactobacillus* LTA on the host’s immune system, we constructed the LPS-induced RAW 264.7 cell model. *Lactobacillus* LTAs significantly attenuated LPS-induced inflammation, which was reflected by a decreased production of cytokines TNF-α and IL-6 and an increased production of IL-10. In accordance with other reports [29], the phosphorylation of ERK and JNK were dramatically enhanced in LPS-induced RAW 264.7 macrophages (Figure 4). In addition, p65, a subunit of NF-κB, could be activated by LPS treatment (Figure 5), which was coincident with other reports [4]. However, *L. reuteri* LTA inhibited LPS-induced activation of both NF-κB and MAPK (Figure 4 and Figure 5). As a potential therapeutic way, it postulates that LTA triggers MyD88-dependent signaling to attenuate LPS-induced inflammation.

In accordance with other reports [15,16,17], LTAs isolated from all three *Lactobacillus* strains had distinguishable anti-inflammatory properties. Combined with the structure characteristics of LTAs, the distinguishable immunomodulatory activities of *Lactobacillus* might be involved in the distinctive structural features of their LTAs, possibly by affecting the interaction between MAMP and PRR, ultimately modulating cytokine expression through a signaling cascade. However, through the evaluation of the in situ role of LTA in live bacteria, LTA mutants have protective effects on inflammation in lactobacilli. An LTA-deficient strain of *L. acidophilus* NCFM deleted for phosphoglycerol transferase significantly improved anti-inflammatory activity in mouse models for IBD [30]. In *L. rhamnosus* GG, a *dltD* mutant carrying a modified LTA structure showed a significantly decreased expression of cytokine IL-8 in the Caco-2 cell line; moreover, it also showed an elevated probiotic activity in a DSS-induced mice colitis model [1,31]. In *L. plantarum* NCIMB8826, a *dlt* mutant deficient in the D-alanylation of teichoic acids enhanced anti-inflammatory capacity and played more protective roles in a murine colitis model [27]. All observations corroborated the notion that LTA modification could be superior in alleviating colitis in lactobacilli, showing the genetic diversity and complexity of the intestinal niche. Conversely, it was coincident with other reports that LTA-mediated immunomodulation could trigger proinflammatory responses in certain individuals, such as those with severely progressed colitis but not a healthy state due to complex microbial communities and host-related mechanisms [1].

## 5. Conclusions

LTA has gained interest as a major MAMP and is an important effector molecule in lactobacilli. *Lactobacillus* LTA shows strong anti-inflammatory activity in the LPS-induced macrophage model through the MAPK and NF-κB signal pathways. The findings indicated that LTA-mediated *Lactobacillus*–host interaction was an important factor to take into account when investigating immunomodulatory effects, which deserves further consideration as an alternative therapeutic for UC treatment.

## Data Availability

The data used in this study are available in this article.
